# A Comparison of White and Yellow Seminal Plasma Phosphoproteomes Obtained from Turkey (*Meleagris gallopavo*) Semen

**DOI:** 10.3390/ijms25189941

**Published:** 2024-09-14

**Authors:** Katarzyna T. Rafalska, Aleksandra Orzołek, Joanna Ner-Kluza, Paweł Wysocki

**Affiliations:** 1 Department of Animal Biochemistry and Biotechnology, Faculty of Animal Bioengineering, University of Warmia and Mazury in Olsztyn, Oczapowskiego 5, 10-719 Olsztyn, Poland; kasia.rafalska13@gmail.com (K.T.R.); pawel.wysocki@uwm.edu.pl (P.W.); 2 Department of Biochemistry and Neurobiology, Faculty of Materials Science and Ceramics, AGH University, Mickiewicza 30, 30-059 Kraków, Poland; nerjoanna@gmail.com

**Keywords:** seminal plasma, yellow semen syndrome, biochemical parameters, phosphoproteome, turkey

## Abstract

Seminal plasma is rich in proteins originating from various male reproductive organs. The phosphorylation of these proteins can significantly impact sperm motility, capacitation, and acrosome reaction. Phosphoproteomics identifies, catalogues, and characterizes phosphorylated proteins. The phosphoproteomic profiling of seminal plasma offers valuable insights into the molecular mechanisms that influence semen quality and male fertility. Thus, the aim of this study was a phosphoproteomic analysis of white and yellow turkey seminal plasma. The experimental material consisted of 100 ejaculates from BIG-6 turkeys between 39 and 42 weeks of age. The collected white and yellow turkey seminal plasmas were analyzed for total protein content; the activity of selected enzymes, i.e., alkaline phosphatase (ALP), acid phosphatase (ACP), superoxide dismutase (SOD), glutathione peroxidase (GPx), and catalase (CAT); and the content of reduced glutathione (GSH) and malondialdehyde (MDA). Phosphoproteins were isolated from white and yellow seminal fluids, and the resulting protein fractions were separated by SDS-PAGE and Western blotting. Phosphorylated residues were immunodetected, and the isolated phosphoproteins were identified (nano LC-MS/MS). Yellow seminal plasmas were characterized by higher levels of total protein, GSH, and MDA, as well as higher levels of ALP, ACP, and GPx activity. There were no significant differences in the activity of SOD and CAT. A total of 113 phosphoproteins were identified in turkey seminal fluids. The functional analysis demonstrated that these phosphoproteins were mainly involved in oocyte fertilization, organization and metabolism of the actin cytoskeleton, amplification of the intracellular signal transduction pathway, general regulation of transport, vesicular transport, proteome composition of individual cellular compartments, and the organization and localization of selected cellular components and macromolecules. Increased phosphorylation of the fractions containing proteins encoded by SPARC, PPIB, TRFE, QSOX1, PRDX1, PRDX6, and FASN genes in white plasmas and the proteins encoded by CKB, ORM2, APOA1, SSC5D, RAP1B, CDC42, FTH, and TTH genes in yellow plasmas was observed based on differences in the optical density of selected bands. The obtained results indicate that the phosphorylation profiles of turkey seminal plasma proteins vary depending on the type of ejaculate.

## 1. Introduction

Yellow semen syndrome (YSS) is a significant problem that affects commercial male turkeys [1]. Normal semen is white, whereas a yellow color characterizes YSS semen. In addition to the altered color, the quantitative and qualitative parameters of the ejaculates are reduced, which can undermine fertilization and hatching success [2,3,4,5]. Elevated total protein content in seminal plasma is a crucial indicator of YSS [2,3,4]. In white semen, the protein content of the seminal plasma does not exceed 10 mg/mL, but it exceeds 20 mg/mL in the seminal plasma of turkeys with YSS. The levels of cholesterol, albumin (ALB), transthyretin, hemopexin, immunoglobulins, and proteinase-like protein can be used as potential protein markers of the YSS. All of the above parameters were elevated in YSS turkeys [1].

Avian seminal plasma mainly consists of epididymal fluid and components that pass directly from the blood, because there are no additional sex glands in the reproductive system of birds. The composition of seminal plasma may be influenced by various factors, including nutrition and age [6,7,8]. Seminal plasma components affect sperm functionality, including the spermatozoa’s ability to interact with secretions of the female reproductive system and to transmit cellular signals that modulate immunological phenomena [9]. Seminal plasma proteins influence most cellular functions, including metabolism, immunity, regulation of oxidation-reduction processes, proteolysis, apoptosis, ion homeostasis, and antimicrobial defenses. The variable amount of proteins is associated with differences in the fertilizing capacity of poultry sperm [10]. The interactions between seminal plasma and sperm induce metabolic changes, binding of seminal proteins to the surface of sperm cells, and membrane remodeling. These processes affect sperm transport and storage in the female genital tract, as well as zygote formation [11].

Phosphorylation is a post-translational modification that regulates the function and properties of proteins. It is estimated that up to 30–65% of cell proteins may undergo phosphorylation reactions, and some proteins undergo phosphorylation multiple times [12]. Kinases usually attach a phosphate group to one of the three amino acid residues (serine, threonine, and tyrosine), whereas phosphatases catalyze dephosphorylation. The activity of kinases and phosphatases allows cells to remain in homeostasis. These enzymes regulate cell metabolism by activating and deactivating selected enzymes [13]. The phosphoproteome of avian sperm has not been thoroughly investigated to date, and it remains poorly understood. However, the effect of phosphorylation/dephosphorylation on sperm motility and the activity of the kinases and phosphatases responsible for these reactions have previously been demonstrated [14,15]. Hence, this study aimed to investigate turkey seminal plasma’s phosphoproteome and reveal potential differences in the plasma protein profiles of white and YSS semen.

## 2. Result

### 2.1. Biochemical Parameters of White and Yellow Seminal Plasmas Derived from Turkey Semen

A higher content of total protein, GSH, and MDA, and a higher ALP, ACP, and GPx activity characterized the yellow seminal plasmas. No significant differences were found in the activity of SOD and CAT (Table 1).

### 2.2. Identification of White and Yellow Seminal Plasma Phosphoproteins by Mass Spectrometry

**Figure 1:**
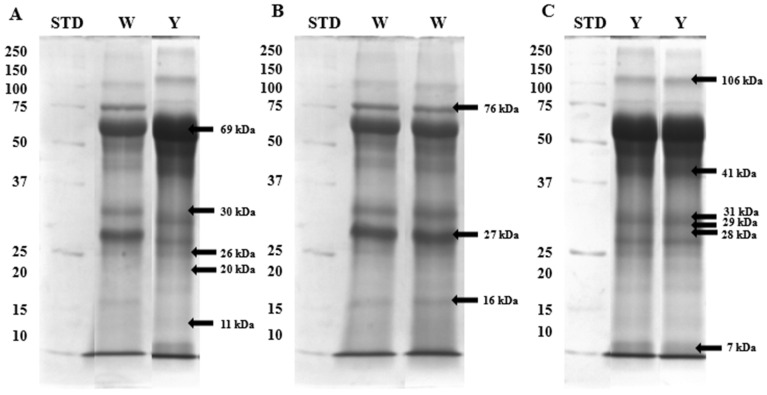
Specific phosphoproteins demonstrated by SDS-PAGE in both white (W) and yellow (Y) seminal plasma (**A**), white seminal plasma (**B**), and yellow seminal plasma (**C**) of turkeys.

Regardless of semen type, the electrophoretic profiles of all samples demonstrated five proteins with a molecular mass of 69, 30, 26, 20, and 11 kDa (Figure 1A). Protein fractions with a molecular weight of 76, 27, and 16 were more abundant in electropherograms of white seminal plasmas (Figure 1B), whereas proteins with a mass of 106, 41, 31, 29, 28, and 7 kDa were more expressed in yellow seminal plasmas (Figure 1C).

Figure 1 shows the fractions excised from SDS-PAGE gels and subjected to the nano LC-MS/MS analysis based on the mean OD values (Table 2), marked with arrows. The results of the mass spectrometry identification are presented in the Supplementary Materials (Table S1).

### 2.3. Immunodetection of White and Yellow Seminal Plasma Phosphoproteins

**Figure 2:**
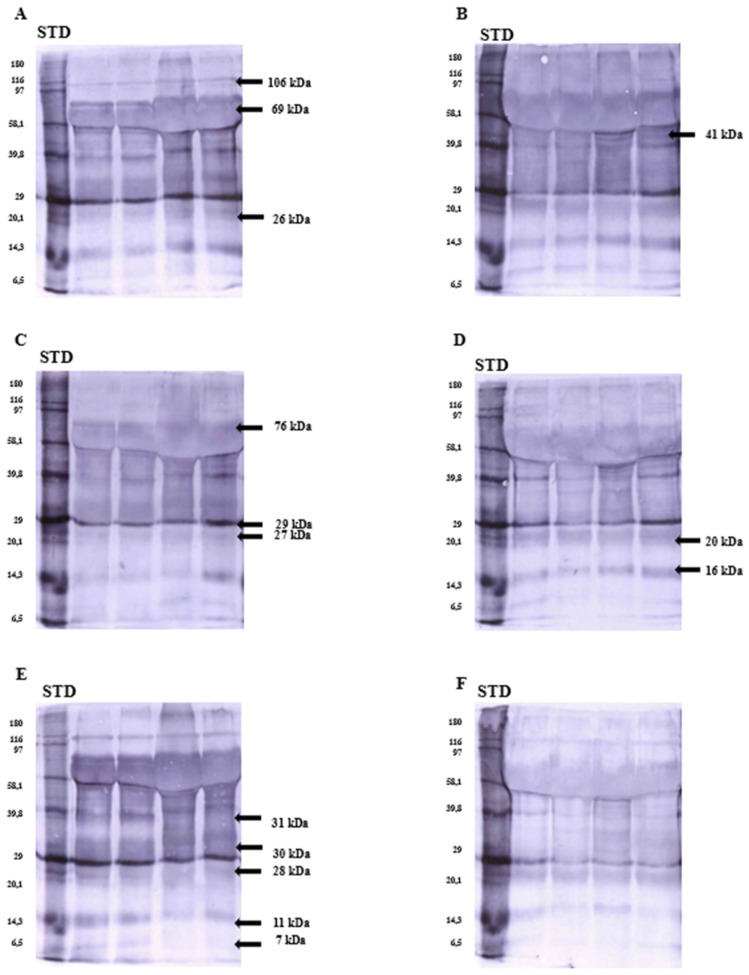
Immunodetection of fractions in white (**A**,**C**,**E**) and yellow (**B**,**D**,**F**) seminal plasmas phosphorylated on different residues, i.e., serine (**A**,**B**), threonine (**C**,**D**), and tyrosine (**E**,**F**).

Western blotting and immunoblotting analyses were conducted to identify the canonically phosphorylated residues of the proteins previously mentioned and assess how these residues’ phosphorylation levels varied between the two types of ejaculate. The phosphorylation profiles of seminal plasma proteins from white and yellow ejaculates were partly convergent, especially regarding low-molecular-weight phosphoproteins. Western blotting and immunoblotting confirmed the presence of phosphoproteins with molecular weights of 106, 76, 69, 41, 31, 30, 29, 28, 27, 26, 20, 16, 11, and 7 kDa (Figure 2). The optical density analyses of stained fractions revealed that the phosphorylation of tyrosine residues was more intensified in white semen. Significant differences in the optical densities of fractions phosphorylated on tyrosine residues were found in the case of 76, 41, 31, 30, 29, 28, 27, 26, 11, and 7 kDa proteins (Table 3). In addition, polypeptide with a molecular mass of 76 kDa was characterized by a significantly higher phosphorylation rate of threonine residues (Table 3). Three proteins of 41, 20, and 16 kDa were subjected to more robust phosphorylation on serine and threonine residues in yellow seminal plasma, although in a non-significant manner. Furthermore, two proteins, with molecular weights of 106 and 69 kDa, showed statistically significant differences in the optical density of the stained bands for all three types of identified residues (Table 3).

### 2.4. The Results of Functional Analysis of the Identified Phosphoproteins

**Figure 3:**
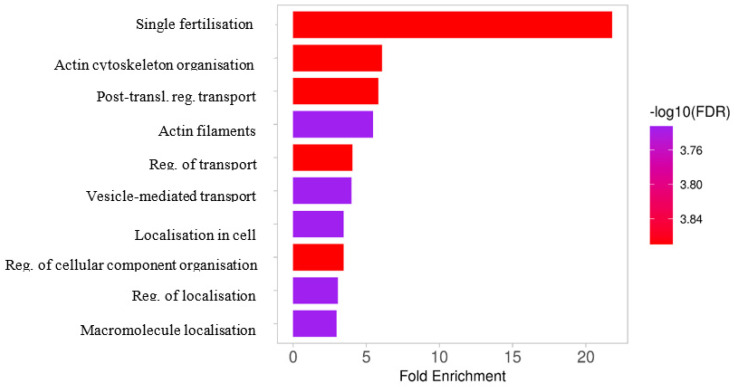
Ten most essential functions of the identified turkey seminal plasma phosphoproteins. Based on the GO Biological Process database in ShinyGO 0.77.

**Figure 4:**
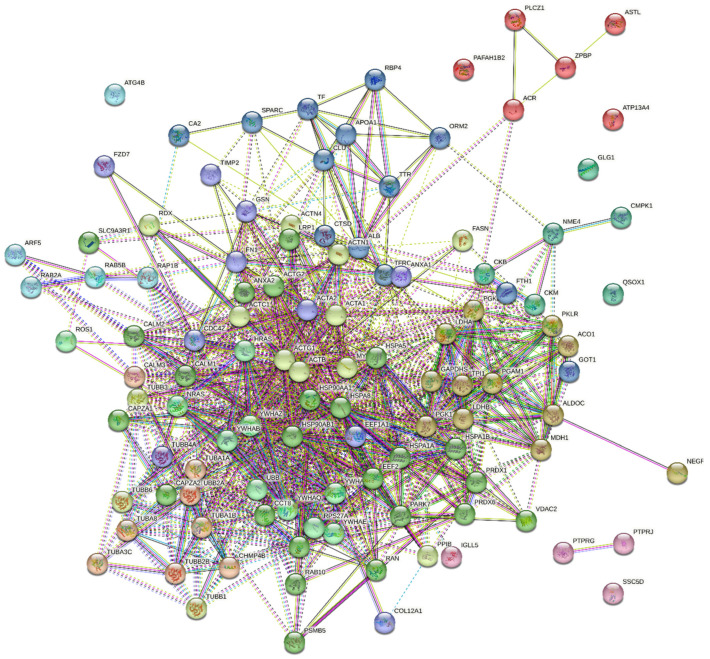
An analysis of the network protein–protein interactions between the phosphoproteins identified in turkey seminal plasma based on the STRING 11.5 database. The proteins were divided into 11 functional groups. The lines connecting individual proteins represent the interactions. The types of interactions are marked with differently colored lines: turquoise—known interactions that were identified in selected databases, pink—known interactions that were determined experimentally, green—interactions predicted based on gene neighborhood, red—interactions that are likely on the assumption of gene fusion, blue—interactions predicted based on the co-occurrence of genes in metabolic pathways, yellow—data from the database, black—gene co-expression, purple—protein homology.Dashed lines represent interactions between proteins from different groups.

**Figure 5:**
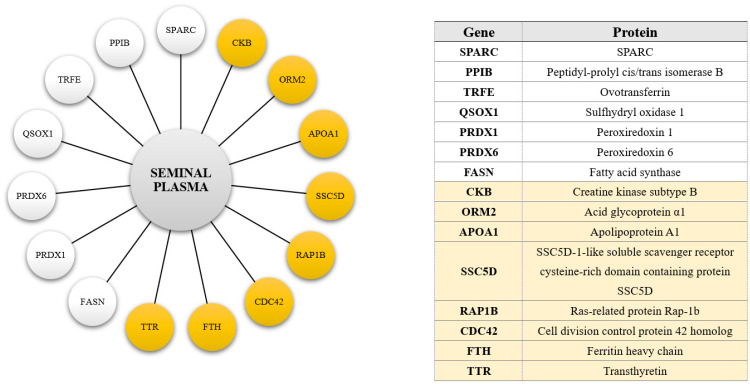
Genes encoding some proteins present in bands differentially phosphorylated in white (white circles) and yellow (yellow circles) seminal plasmas.

A total of 113 phosphoproteins were identified in turkey seminal plasma. The detected phosphoproteins were mainly involved in oocyte fertilization, organization and metabolism of the actin cytoskeleton, amplification of the intracellular signal transduction pathway, general regulation of transport, vesicular transport, the proteome composition of individual cellular compartments, and the organization and localization of selected cellular components and macromolecules (Figure 3). An analysis of protein-protein interactions revealed that seminal plasma proteins (Figure 4) formed a network of proteomic connections divided into 11 clusters. Figure 5 shows the genes encoding proteins present in bands differentially phosphorylated in white and yellow seminal plasmas.

Cluster 1 included proteins encoded by ALB, APOA1, CA2, CLU, CTSD, FTH1, GOT1, ORM2, RBP4, SPARC, TF, TFRC, and TTR genes. Albumin (ALB) entered into the most significant interactions with APOA1 (0.987), GOT1 (0.742), ORM2 (0.848), RBP4 (0.825), SPARC (0.989), TF (0.836), TFRC (0.801), and TTR (0.943). Apolipoprotein 1 (APOA1) interacted with CLU (0.995), ORM2 (0.830), RBP4 (0.788), TF (0.976), and TTR (0.975). The interactions in cluster 1 occurred between the dominant seminal plasma proteins, seminal iron, and lipid regulatory proteins. Strong interactions were also found between iron management proteins FTH1 and TFRC (0.989), and TF and TFRC (0.999). Cluster 2 comprised proteins encoded by ACO1, ALDOC, GAPDHS, LDHA, LDHB, MDH1, NEGR1, PGAM1, PGK1, PGK2, PKLR, and TPI1 genes. These proteins are involved in glycolysis. The highest number of interactions was noted between triosephosphate isomerase (TPI1) and ACO1 (0.708), ALDOC (0.982), GAPDHS (0.997), LDHA (0.893), LDHB (0.848), MDH1 (0.798), PGAM1 (0.975), PGK1 (0.999), PGK2 (0.996), and PKLR (0.956). Cluster 3 consisted of proteins encoded by IGLL5, PTPRG, PTPRJ, and SSC5D genes. The protein encoded by PTPRJ dephosphorylates many signaling molecules and is involved in cell adhesion, proliferation, differentiation, and migration. The protein encoded by PTPRG contains a fibronectin domain. No significant interactions between the analyzed proteins were observed in cluster 3. Cluster 4 was composed of proteins encoded by ACR, ASTL, ATP13A4, PAFAH1B2, PLCZ1, and ZPBP genes. These proteins participate in oocyte activation and fertilization. Cluster 4 proteins exhibited moderately strong interactions. Cluster 5 comprised proteins encoded by ARF5, ATG4B, RAB2A, RAB5B, and RAP1B genes. These GTP-binding proteins present GTPase activity and are involved in the endosomal pathway. A strong interaction was observed between the RAP1B and RAB5B proteins of the Ras subfamily. Cluster 6 consisted of proteins encoded by CKB, CKM, CMPK1, GLG1, NME4, and QSOX1 genes. These polypeptides are involved in the biosynthesis of creatine, phosphocreatine, pyrimidine ribonucleotides, and organic phosphates. Three very strong interactions were noted between CKB and CKM (0.974), NME4 and CMPK1 (0.967), and NME4 and CKB (0.789). Cluster 7 comprised proteins encoded by CALM3, CHMP4B, TUBA1A, TUBA1B, TUBA3C, TUBA8, TUBB2A, and TUBB2B genes. These proteins participate in the organization of the cytoskeleton and cell mitosis. Protein 4B of the corpus callosum (CHMP4B, endosome) entered into very strong interactions with the tubulins encoded by TUBA1A (0.901), TUBA1B (0.904), TUBA8 (0.900), TUBB2A (0.900), and TUBB2B (0.900). Calmodulin (CALM3) showed interactions at a much lower confidence level. Cluster 8 contained proteins encoded by HRAS, NRAS, ROS1, RPS27A, UBB, YWHAB, YWHAE, YWHAG, YWHAQ, and YWHAZ genes. These proteins are primarily involved in the apoptotic signaling pathway, and they decrease the risk of DNA mutations. The GTPase encoded by HRAS interacted with NRAS (0.987), ROS1 (0.841), RPS27A (0.919), UBB (0.925), YWHAB (0.941), YWHAE (0.942), YWHAG (0.940), YWHAQ (0.935), and YWHAZ (0.946). Cluster 9 was composed of proteins encoded by ACTA2, ANXA1, CDC42, COL12A1, EEF1A1, FN1, FZD7, GSN, TIMP2, and TUBB4A genes. All of these proteins have a strong affinity for anions and can participate in the organization of the actin cytoskeleton. Fibronectin (FN) interacted with ACTA2 (0.766), ANXA1 (0.944), CDC42 (0.974), COL12A1 (0.778), EEF1A1 (0.973), FZD7 (0.783), GSN (0.942), TIMP2 (0.711), and TUBB4A (0.712). Cluster 10 contained proteins encoded by ACTA1, ACTB, ACTC1, ACTG1, ACTN1, ACTN4, FASN, MYH9, PPIB, RDX, TUBB1, TUBB3, and TUBB6 genes. Most of these proteins form the cell cytoskeleton (actin, actinins, radixin, myosin, tubulin) and enter into very strong interactions. A moderately strong interaction was observed between fatty acid synthase (FASN) and ACTAB (0.624). The interactions between cis-trans peptidyl-proline isomerase B (PPIB), ACTAB (0.571), and ACTG1 (0.452) could suggest that isomerase plays a role in the final formation of the elements that make up the cell motility apparatus. Finally, cluster 11 was composed of 24 proteins encoded by ACTG2, ANXA2, CALM1, CALM2, CAPZA1, CAPZA2, CCT6A, CCT8, EEF2, HSP90AA1, HSP90AB1, HSPA1A, HSPA1B, HSPA5, HSPA8, LRP1, PARK7, PRDX1, PRDX6, PSMB5, RAB10, RAN, SLC9A3R1, and VDAC2 genes. These proteins modulate the activity of cyclic nucleotide phosphodiesterases, phosphoprotein phosphatases, calcineurin, and nitric oxide synthase; influence cell adaptation to heat stress and oxidative stress; and participate in the formation of tubulin agglomerates and caps at the end of actin filaments, protein folding and stabilization, autophosphorylation of proteins, phosphorylation of threonine residues in proteins, sperm binding to the oocyte zona pellucida, and fertilization. The highest number of significant interactions (≥0.07) was observed between the heat shock protein (HSP) family and other proteins. The HSP90AA1 protein participated in the highest number of interactions, and it interacted very strongly (≥0.09) and strongly (≥0.07) with HSPA8 (0.999), HSPA1A (0.999), HSP90AB1 (0, 994), HSPA1B (0.991), HSPA5 (0.978), LRP1 (0.969), CAPZA1 (0.917), CAPZA2 (0.910), CALM2 (0.887), PARK7 (0.871), EE2 (0.829), CCT8 (0.814), CCT6A (0.758), and CALM1 (0.704). HSP family proteins can act as chaperones towards other group 11 polypeptides. HSP family proteins protect these polypeptides, maintain their regulatory functions, ensure uninterrupted intracellular signal transduction, and control the cell cycle.

## 3. Discussion

### 3.1. Differences in Biochemical Parameters Characterising White and Yellow Seminal Plasmas Derived from Turkey Semen

Significant differences were observed in the parameters characterizing white and yellow semen (YSS) in turkeys. These results confirm previous reports of lower total protein [2,3] in white seminal plasma. Hess et al. [16] reported higher levels of AspAT, ACP, and SOD activity in yellow seminal plasma. In the present study, significant differences were noted in the activities of ALP and ACP, which were higher in YSS ejaculates. Bucci et al. [17] demonstrated that ALP activity, mainly manifested in seminal plasma, decreases under capacitating conditions and that adding ALP to spermatozoa during capacitation suppresses this process. This suggests that AP plays a role in keeping spermatozoa inactive until ejaculation and in regulating the development of their fertilizing ability. Therefore, higher ALP activity in yellow seminal plasma may influence the delayed onset of capacitation by YSS sperm. The primary source of AcP in turkeys is the ductal epithelium [18]. The increased ACP activity in seminal plasma may be linked to changes occurring in the turkey reproductive tract or originate from damaged spermatozoa [19]. Hence, such high ACP activity in yellow seminal plasma may have indicated inappropriate functioning of the tom reproductive system. Bucci et al. [17] noted that the activity of ALP also influences the phosphorylation profile of tyrosine residues in sperm proteins. Thus, the higher phosphatases activities observed in our study may have influenced the seminal plasma proteins phosphorylation profile. White and yellow semen did not differ in SOD activity. GPx activity was significantly lower in white semen, while CAT activity was comparable in both types of semen. In a study by Słowińska et al. [20], the activity of SOD, one of the key elements of the antioxidant defense system in semen, was elevated in birds with YSS. Significantly higher GSH and MDA levels were noted in the seminal plasma of birds with YSS, which could indicate that the sperm cells in yellow semen require increased antioxidant protection. Słowińska et al. [21] demonstrated that the significant amounts of malonyl-CoA in YSS plasma may enhance lipid peroxidation. Impaired lipid metabolism may contribute to the formation of numerous fat vacuoles in different segments of the reproductive tract of ejaculate-producing YSS males [22]. A large number of vacuoles may intensify peroxidation processes and the generation of lipid peroxides, which directly increases the demand for antioxidant protection in the seminal plasma of males with YSS. A relationship between oxidative stress-potentiated male infertility and seminal plasma protein profile has been revealed. Recent literature on human infertility has highlighted the link between oxidative stress and the proteomic profiles of seminal plasma [23]. Moreover, additional proteomic studies have shown that oxidative stress can adversely impact semen quality [24]. Thus, the regulation of oxidative stress within semen, which can affect the phosphorylation states of proteins in the seminal plasma, plays a crucial role in maintaining sperm function and overall semen quality.

### 3.2. The Potential Importance of Canonical Phosphorylation in the Physiology of Turkey Semen

The presence of proteins that have undergone abnormal post-translational modification (PTM) has been linked to impaired cell maturation [25]. Some disorders associated with impaired sperm motility have been shown to arise from the deregulation of PTM processes in spermatozoa. In most published studies, attempts were made to determine the extent of canonical phosphorylation resulting from the phosphorylation of serine, threonine, and tyrosine residues in proteins. In turn, amino acids such as histidine, lysine, arginine, glutamic acid, and cysteine undergo non-canonical phosphorylation [26]. The diversity of protein phosphorylation profiles significantly complicates the identification of phosphorylation processes that affect cell viability [27].

In our study, the phosphorylation of tyrosine residues was intensified in most white seminal plasma proteins, whereas the phosphorylation of serine-threonine residues was intensified in yellow seminal plasma proteins. Phosphoserine (pSer) or phosphothreonine (pThr) residues account for more than 90% of the phosphorylated protein residues in a cell. Most proteins in eukaryotic cells strongly favor phosphorylation of Ser over Thr residues. Phosphorylation is induced by the type of kinase substrate (faster phosphorylation of serine than threonine) and by phosphatase activity (faster dephosphorylation of Thr than Ser). However, threonine phosphorylation induces greater overall changes in protein structure than serine phosphorylation. The phosphorylation of serine residues does not cause significant conformational changes in proteins, and it could play a dominant role in processes that require multiple consecutive, stepwise phosphorylation reactions. In contrast, the phosphorylation of Thr residues induces more complex protein structure changes required for rapid functional effects by turning specific protein functions on or off [28]. The phosphorylation of tyrosine residues regulates protein activity in several ways, including inducing electrostatic repulsion and changes in proteins’ chemical affinity for molecules. However, docking is the most important function of phosphotyrosine, which promotes a specific interaction between a protein with phosphorylated tyrosine and another protein that contains a phosphotyrosine-binding domain. These interactions are essential for signal transduction from receptor tyrosine kinases on the cell surface to the cell interior. Kinases activate the binding of the corresponding extracellular ligand and, consequently, trigger a specific cellular response [29]. Increased expression of phosphotyrosine residues in white seminal plasma could indicate that these compounds enhance signal transduction in spermatozoa and prepare the gametes for subsequent stages of activation and fertilization. However, the increased expression of serine and threonine residues in yellow seminal plasma could suggest that these compounds participate in the reorganization of the plasma proteome and are responsible for its final composition.

### 3.3. Proteins of Extracellular Origin Unveiled in the Phosphoproteome of Turkey Seminal Plasma

According to the literature, turkey seminal plasma comprises secretions from the reduced epididymides and vas deferens. However, it can also contain compounds transferred directly from the blood [30]. Many extracellular matrix (ECM) proteins are present in phosphorylated form in seminal plasma. However, little is known about how the phosphorylation of ECM proteins affects the physiology of suspended sperm cells. The extracellular matrix (ECM) plays a crucial role in regulating spermatogenesis, especially in the functioning of spermatogonia; Sertoli cells; and maintaining the blood–testis barrier. The restructuring of junctions at the blood–testis barrier (BTB) in the seminiferous epithelium during spermatogenesis appears to be regulated by complex interactions involving cytokines (e.g., TNFα), proteases (MMP-2, MMP-9, MT1-MMP), protease inhibitors (TIMP-1, TIMP-2), collagens, laminins, adaptors, kinases, and phosphatases [31]. Numerous mass spectrometry studies have shown that some ECM proteins, including vitronectin, laminin-1, osteopontin, sialoprotein, collagen, and fibronectin, can exist in phosphorylated form [32]. The present study demonstrated that turkey seminal plasma contains albumin, ovotransferrin, fibronectin, transthyretin, collagen chains, and immunoglobulins classified as of extracellular origin that undergo phosphorylation reactions. The phosphorylation of specific amino acid residues may affect the biological activity of these proteins. Martin and Ekman [33] reported that the phosphorylation of plasma ALB depends on its site of origin and animal species. Visconti et al. [34] observed a correlation between the ALB content of the medium and the phosphorylation of tyrosine residues in stored spermatozoa. They suggested that the ALB-induced cholesterol efflux from the plasma membrane is correlated with the cAMP-induced phosphorylation of tyrosine residues in spermatozoa. In turn, the phosphorylation of other ECM proteins, such as ovotransferrin, may alter their structure and improve their thermostability and emulsifying properties [35]. Fibronectin has binding domains for other proteins, including collagen, fibrin, heparin, DNA, and actin. The mentioned protein in a phosphorylated state promotes cell attachment and enhances mechanical cell functions [32]. An ESI-MS analysis revealed that transthyretin (TTR), otherwise known as prealbumin, mainly occurs in plasma in the cysteine-conjugated form (Cys), but TTR derivatives with a higher molecular weight of 32 Da (dihydroxylation), 80 Da (phosphorylation), and 306 Da (glutathionylation) were also identified [36]. It belongs to the so-called acute phase proteins (BOFs). TTR is a negative BOF, meaning its amount in the body decreases during the inflammatory process. In avian blood, transthyretin shows triiodothyronine (T3) and thyroxine (T4) binding properties. Phosphoproteomics studies have also confirmed the phosphorylation of serine (Ser), threonine (Thr), and tyrosine (Tyr) residues in collagen chains. Phosphorylation and dephosphorylation processes are believed to play a regulatory role in collagen processing, stability, assembly, degradation, and binding [37]. Although post-translational modifications in immunoglobulin chains have not yet been demonstrated to involve phosphorylation, we cannot completely rule out this possibility. The presence of the mentioned proteins in almost every band obtained after electrophoresis and subjected to identification can be explained by the enormous amount of these polypeptides in the turkey semen.

### 3.4. Parts of the Ubiquitinating System among the Phosphoproteins Identified in Turkey Seminal Plasma

In addition to phosphorylation, sperm proteins undergo ubiquitination during sperm maturation in the epididymides. This post-translational change plays a crucial role in many biological processes, by eliminating defective spermatozoa (mainly those with morphological defects) through phagocytosis [38]. The ubiquitination-specific degradation system is also involved in the gamete formation cycle during the first two phases of spermatogenesis and the remodeling of haploid spermatids. Ubiquitination is essential in spermatogenesis when protamines replace histones [39]. The proteins associated with degradation processes in the protein ubiquitination pathway, including ubiquitin ligases (UBR4 and HUWE1) and polyubiquitin (UBB), as well as ribosomal ubiquitin-40S proteins such as ribonucleoproteins S27a and RPS27A, were found to be hyperphosphorylated in spermatids with low motility. Phosphorylation processes were also differentiated in Ras-related proteins (such as RAB2A, RAB2B, or RAB4A) that participate in vesicular transport, and in ribosomal proteins (such as RPLP2, RPL15, or DAP3) that are involved in protein metabolism [40]. Proteins of the RAB family regulate the stages of vesicular transport and facilitate the binding of cytoskeleton proteins to sperm plasma membranes [41]. In the current study, the turkey semen contained polyubiquitin and the RPS27A protein, which are most likely involved in the apoptosis of defective and/or morphologically abnormal or malfunctioning spermatozoa. Several RAB factors, including RAB2, RAB5, and RAB10, were also identified in the turkey seminal plasma, which could be attributed to the fact that these factors participate in the organization and activation of the sperm motility mechanisms.

### 3.5. Components of Protein Folding/Unfolding Apparatus Present in the Phosphoproteome of Turkey Seminal Plasma

Heat shock proteins are potentially linked to semen quality, because they are essential for normal physiology, spermatogenesis, and sperm maturation [42]. The overarching role of HSPs is to control protein folding or refolding in misfolded polypeptides and to control the targeting of tagged proteins for subsequent degradation. Hence, reduced HSP expression may be associated with decreased sperm motility due to the accumulation of misfolded proteins [43]. These proteins are often produced in response to cellular stress and are likely to be overexpressed in repairing heat stress damage [44]. When cells are exposed to a heat stressor, HSP70 increases SOD activity to protect cells against oxidative damage. HSP70 regulates SOD activity to protect sperm plasma membranes against reactive oxygen species (ROS). There is evidence to suggest that HSP70-2 could participate in the regulation of spermatozoa’s thermotolerance of acute or chronic temperature changes [45]. Martin-Hidalgo et al. [46] also found that HSPB1 and HSP90A were phosphorylated differently in spermatozoa subpopulations characterized by high and low motility. In turkey semen, HSPA5, HSPA8, HSPA9, HSP70, and HSP90 most probably act as chaperones for the newly formed sperm proteins. These proteins could be overexpressed in turkey semen because they protect the gametes from the adverse effects of a changing environment. Phosphorylation could affect the biological activity of these proteins or their affinity for sperm.

### 3.6. The Phosphoproteome of Turkey Seminal Plasma Includes Some Regulatory Proteins

The phosphorylation profiles of proteins that participate in carbohydrate metabolism and energy production, including fructose-bisphosphate aldolase A (ALDOA), glyceraldehyde-3-phosphate dehydrogenase (GAPDH), and mannose-6-phosphate isomerase (MPI), are diverse in sperm subpopulations with varied motility. MPI is hypophosphorylated in spermatozoa with low motility, whereas the intensity of ALDOA and GAPDH phosphorylation is higher [40]. Furthermore, higher levels of triosephosphate isomerase (TPI), a highly efficient metabolic enzyme that participates in glycolysis and gluconeogenesis, as well as both isoforms of phosphoglycerate mutase (PGAM1 and PGAM2), are associated with reduced sperm motility [47]. Some of them have been identified as phosphoproteins present in turkey seminal plasma. The degree to which these proteins are phosphorylated may affect their biological functions and, consequently, sperm metabolism.

The lipid composition of the sperm plasma membrane is an important factor that influences sperm functionality and integrity. Different types of lipids are involved in each key step of the egg fertilization process. Lipid metabolism has to be tightly regulated because it is responsible for the key physiological processes in sperm cells, such as the acquisition of motility, capacitation, acrosome reaction, and sperm–oocyte fusion. Research has shown that a sperm cell’s fertilizing capacity largely depends on membrane lipid homeostasis [48]. Annexin A2 (AnxA2) is a versatile protein that plays a role in various cellular functions, including endocytosis, exocytosis, membrane domain organization, lipid reorganization, actin remodeling, signal transduction, protein assembly, transcription, mRNA transport, and DNA replication and repair. ANXA2 is a calcium-regulated phospholipid-binding protein that is mainly found in exosomes that target and fuse exosomes. Aberrant expression of ANXA2 has been observed during abnormal ubiquitination processes. Munuce et al. [49] observed lower levels of ANXA1 and ANXA2, and higher levels of the corresponding mRNA, in low-quality semen, whereas Martins et al. [50] demonstrated overexpression of ANXA2 in the seminal plasma of men with primary and secondary infertility. In turn, the overexpression of phosphoglycerate kinase (PGK), outer dense fiber (ODF) protein, clusterin (CLU), voltage-dependent anion-selective channel 2 (VDAC2), and zona pellucida binding protein (ZPBP) has been linked to DNA fragmentation, and they have been identified as potential biomarkers for assessing semen quality [51]. In our study, phosphorylated forms of all of the above proteins were identified in turkey seminal plasma. The key phosphorylation sites in ANXA2 are Ser11, Ser25, and Tyr23. Phosphorylation at Ser25 is associated with annexin’s binding to secretory granules, while phosphorylation at Tyr23 is necessary for its interaction with actin, early endosomes, and multivesicular bodies (MVBs). The second modification allows annexin to enter the lumen of exosomes and localize to the extracellular cell surface [52]. The phosphorylation of selected proteins may be affected by the physiological status of spermatozoa. For example, VDAC2, F-actin cap protein, ODF, and α-tubulin have been shown to undergo increased phosphorylation of tyrosine residues during sperm capacitation [53].

### 3.7. The Differences in White and Yellow Turkey Seminal Plasma Phosphoprotein Profiles

Higher expression of tyrosine residues in bands containing SPARC, peptidyl-prolyl cis/trans isomerase B (PPIB), ovotransferrin (TRFE), sulfhydryl oxidase 1 (QSOX1), peroxiredoxin 1 (PRDX1), peroxiredoxin 6 (PRDX6), and fatty acid synthase (FASN) was noted in white seminal plasmas.

The presence of certain proteins has been linked to ECM development. These proteins include SPARC, PPIB, and QSOX1, whose phosphorylated forms were observed in the present study. Both the conformation and the activity of many proteins phosphorylated on serine and threonine residues are regulated by isomerization of phosphorylated Ser/Thr-Pro bonds. SPARC belongs to a family of proteins that interact with the cell matrix and modulate cell adhesion. This highly conserved protein is found in the semen of many mammalian species [54]. SPARC exhibits anti-adhesive activity, which is mediated by a pathway dependent on the phosphorylation of tyrosine residues [55]. After the cleavage of the signaling sequence, SPARC becomes a 32 kDa protein, but the secreted form is identified as a 43 kDa protein when the glycoprotein residue is attached to the original structure [56]. Higher phosphorylation of tyrosine residues in a band containing SPARC may indicate its improved anti-adhesion ability. In the current study, this protein was identified based on several bands on SDS-PAGE gels, which could suggest that it contains a glycosidic residue and multiple disulphide bonds and is broken down into derivatives with different molecular weights. Peptidyl-prolyl cis-trans isomerase B (PPIB) catalyzes the cis-trans isomerization of peptidyl-prolyl peptide bonds, which is preceded by the phosphorylation of serine or threonine residues. The PPIB gene is highly expressed in Sertoli cells, and its deactivation points to a disruption of the blood–nucleus barrier [57]. Sulfhydryl oxidase 1 (QSOX1) is involved in protein folding, maintenance of homeostasis in the endoplasmic reticulum, and regulation of the cell cycle [58]. In semen, QSOX1 is found mainly in seminal plasma, and it undergoes phosphorylation on serine, threonine, and tyrosine residues. QSOX1 probably causes agglutination of spermatozoa that are susceptible to oxidative stress and apoptosis [59]. In turkey semen, enhanced phosphorylation of QSOX1 could facilitate the elimination of damaged spermatozoa, increase ejaculate quality, and minimize the adverse effects of oxidative stress. Ovotransferrin (TRFE) is present in high concentrations in the seminal plasma of cockerels and turkeys [8,23]. TRFE exhibits bactericidal activity in the egg yolk, and it has been suggested to have similar activity in poultry seminal plasma. Ovotransferrin does not occur naturally in mammalian semen; therefore, high concentrations of this protein in the avian reproductive system are probably responsible for maintaining specific immune functions and for protecting birds’ secretions from pathogens [10]. The fact that ovotransferrin was more highly phosphorylated in white semen may indicate its greater antimicrobial and protective effects. Peroxiredoxins (PRDX) are a family of antioxidant enzymes that control ROS levels in sperm. PRDX6 plays a major role in neutralizing ROS to maintain sperm viability and motility and to enable sperm to achieve fertilizing capacity [60]. PRDX1 associated with membranes undergoes temporary phosphorylation at tyrosine-194, resulting in its inactivation, both in cells stimulated through growth factor or immune receptors in vitro. This localized PRDX1 inactivation facilitates the transient buildup of H_2_O_2_ near membranes, where signaling molecules are concentrated, while preventing harmful H_2_O_2_ accumulation in other areas [61]. Peroxiredoxin 6 (Prdx6) functions as a Ca^2+^-independent intracellular phospholipase A2 (referred to as aiPLA2) and is found in the cytosol, lysosomes, and lysosome-related organelles. Its activity is low at cytosolic pH but increases substantially upon enzyme phosphorylation. Its primary roles include repairing peroxidized cell membranes and activating NADPH oxidase type 2 (NOX2) [62]. In the present study, strongly phosphorylated bands containing PRDX1 and PRDX6 were identified in white seminal plasma, indicating that these enzymes provide effective protection against oxidative stress. Fatty acid synthase (FASN) occurs in turkey tissues as a dimer with a molecular weight close to 250 kDa. FASN is a circulating biomarker, and its activity inside the cell is inversely correlated with its expression in extracellular fluid [63]. For example, very low levels of FASN activity in spermatozoa have been found to increase the fragmentation of sperm DNA, while above-normal expression of this enzyme in semen has been linked to early pregnancy loss [64]. Phosphorylation of tyrosine residues in FASN is crucial for maintaining its biological activity in certain cell types [65]. Therefore, the elevated phosphorylation of the FASN-containing band may suggest a need for biological protection of the enzyme’s activity, while also indicating enhanced enzymatic efficiency.

In the present study, seminal plasmas with YSS were characterized by a higher abundance of bands, phosphorylated on serine and threonine residues, containing creatine kinase type B (CKB), α-1-acid glycoprotein-like protein (ORM2), apolipoprotein A1 (APOA1), SSC5D-1-like soluble scavenger receptor cysteine-rich domain-containing protein (SSC5D), Ras-related protein Rap-1b (RAP1B), cell division control protein 42 homolog (CDC42), and ferritin heavy chain (FTH). Creatine kinase (CK) plays a crucial role in sperm function by catalyzing energy regeneration through the shuttle system. High-energy production in sperm is compartmentalized, with two distinct creatine kinase isoenzymes located in the sperm tail and the mitochondria-rich midpiece region [66]. The inactivation of CK can disrupt sperm motility patterns [67]. In turn, high CK activity is associated with poor sperm quality [68]. Research has demonstrated that CK-B undergoes post-translational modification by an unidentified protein kinase. However, the function of this modification remains unclear [69]. So far, it has only been demonstrated that protein kinase (AKT), activated by insulin-like growth factor 1 receptor signaling, phosphorylates creatine kinase B (CKB) at T133, leading to a decrease in CKB’s metabolic activity and an increase in its binding to glutathione peroxidase 4 (GPX4) [70]. The stronger Ser-Thr phosphorylation of CKB in the yellow seminal could be indicative of inferior ejaculate quality, due to impaired sperm maturation. The Orm family proteins are conserved integral membrane proteins located in the endoplasmic reticulum and serve as crucial regulators of sphingolipid biosynthesis homeostasis. Alpha-1-acid glycoprotein (ORM2) has immunomodulatory effects as an acute-phase protein produced in response to systemic inflammatory reactions. The ORM2 protein is one of 21 polypeptides whose concentration increases in seminal plasma characterized by considerable fragmentation of sperm DNA [71]. A higher percentage of spermatozoa with defragmented DNA in this type of semen could explain the presence of phosphorylated ORM2 in the ejaculates of individuals with YSS. In yeast, ORM protein phosphorylation is needed for the initiation of the sphingolipid biosynthesis pathway; however, in animals, such a cycle has not been demonstrated to date. A protein with an estimated molecular weight of 250 kDa, isolated from seminal plasma by FPLC chromatography and SDS-PAGE electrophoresis, was found to influence the activation of sperm motility. An analysis of its amino acid sequence revealed the presence of three components: apolipoprotein A1, and immunoglobulin heavy and light chains. The protein’s ability to activate sperm motility was inhibited by its degradation, which suggests that the interactions between its components enhance sperm motility. Attempts have been made to activate sperm motility through individual components, but none have exerted such spectacular effects [72]. It has been reported that APOA1 induces cholesterol efflux from spermatozoa. Moreover, this seminal plasma protein influences sperm binding to the oocyte and, ultimately, male fertility [73]. Different concentrations of APOA1 were found in the seminal plasma of male chickens characterized by varied fertilizing capacity, which validates the observation that the lipoprotein complex in seminal plasma plays a key role in fertility outcomes [74]. In the present study, both APOA1 and immunoglobulin chains were identified in turkey semen. Although no interactions between these proteins were found, the fact that they occurred simultaneously in many bands might suggest otherwise. More pronounced phosphorylation of Ser-Thr residues of APOA1 in yellow seminal plasma could affect the enzyme’s activity and cause uncontrolled cholesterol leakage from the sperm, thus impairing sperm function. The cysteine-rich scavenger receptor (SRCR) family comprises a group of proteins that are attached to the cytoplasmic membrane or are secreted extracellularly. Although no complex biological function has been attributed to the SRCR family, some of these receptors have been shown to recognize the pathogen-associated molecular patterns (PAMPs) of bacteria, fungi, and other microorganisms. SSC5D is a soluble SRCR receptor produced by monocytes, macrophages, and T lymphocytes [75]. The presence of SSC5D in yellow ejaculates could be indicative of the presence of macrophages or lymphocytes and increased demand for protection against external factors. The proteomic databases report that SSc5d contains a phosphorylated residue at position S919, but its role is not yet known. Rap1 is expressed in differentiating spermatids, and it is believed to take part in the process of sperm differentiation and the regulation of germ cell–Sertoli cell adhesion [76]. Phosphorylation of RAP1B is required for cAMP-dependent mitogenesis, tumorigenesis, and inhibition of AKT activity [77]. The mentioned authors suggested that phosphorylation either directly “fixes” a conformational state or indirectly alters the interstate exchange rates. Being subjected to increased phosphorylation at the serine and threonine residues in turkey yellow seminal plasma might have influenced the type and/or mode of action of the RAP1B protein. The same could apply to the actioning of the CDC42 homolog. This protein is required for steady-state spermatogenesis, fetal and early postnatal testis development, proper cell polarity, and blood–testis barrier function [78]. The phosphorylation states of CDC42 regulate its interactions with RhoGDI and, consequently, its extraction from biological membranes [79]. The mentioned authors suggested that phosphorylation is a mechanism of regulation of CDC42 activity, independently of GDP-GTP cycling. Ferritin heavy chain (FTH) plays a key role in maintaining cellular iron homeostasis, regulating the oxidative stress response, cell apoptosis, ovarian follicle development, and other physiological processes [80]. Disturbances in cellular FTH levels contribute to the pathogenesis of inflammation and/or prolonged infection. FTH contains many serine and threonine residues that are readily phosphorylated in its structure. The phosphorylation of ferritin residues has been linked to the nuclear translocation of FTH. This process may be partly blocked when FTH phosphorylation is inhibited. Nuclear ferritin has been shown to protect DNA from UV- or iron-dependent DNA damage [81]. The present study identified a phosphorylated FTH in turkey seminal plasma. In turkeys with YSS, the phosphorylation of FTH on serine and threonine residues may have been intensified and thereby influenced its activity, which may have been insufficient. Transthyretin (TTR) is primarily responsible for transporting thyroxine and the retinol-binding protein (RBP) complex to various parts of the body and brain. TTR is a serum protein that consists of four 14 kDa monomers in association with RBP (1:1), which are transported to peritubular myoid cells and passed into Sertoli and spermatogenic cells, bypassing the blood–nucleus barrier [82]. Because TTR concentration correlates positively with the levels of ROS and reactive nitrogen species (RNS) [83], it can induce oxidative stress in the endoplasmic reticulum [84]. The rise in phosphorylation levels is, for example, more pronounced in turpentine-injected rats, indicating that the observed increase may be linked to inflammation pending in vivo [85]. Increased phosphorylation of TTR may, therefore, suggest that pro-oxidative processes are intensified in the seminal plasma of turkeys with YSS.

This study made the first attempt to identify and characterize the phosphoproteome of turkey seminal plasma. A significant part of the phosphoproteins identified in our study had previously been reported in turkey seminal plasma, including those encoded by genes such as ALB, TRFE, COL12A1, QSOX1, ASTL, APOA1, GSN, ORM2, SPARC, ANXA2, PLCZ1, TPI1, FTH1, MDH1, LDHA, LDHB, and ALDOC [22]. Phosphorylated forms of the identified proteins have also been found in the semen of many mammalian species, including humans. In the literature, some of these phosphoproteins have been identified as potential biomarkers of ejaculate quality and/or sperm fertilizing capacity. The analyzed group of identified turkey seminal plasma phosphoproteins include proteins that participate in sperm maturation, the development of the sperm motility apparatus, cellular metabolism, protection against oxidative stress, and egg fertilization.

## 4. Materials and Methods

The experimental material consisted of 100 turkey (*Meleagris gallopavo*) ejaculates collected from BIG-6 line males (Aviagen, Huntsville, AL, USA) between 39 and 42 weeks of age. The birds were raised on a commercial farm (GERCZAK Nord-Pol Hatchery, Kozia Góra, Province of Warmia and Mazury), which adheres to the highest hygiene and biosafety standards. Semen was collected using the method proposed by Burrows and Quinn [86]. Semen samples collected from each bird were placed in separate syringes and classified as white (n = 50) or yellow (n = 50). A qualified farm employee assessed semen color against a blue background and assigned each ejaculate to one of these quality groups. Ejaculates with a white, milky, or pearl color were categorized as white (W). In contrast, those with a yellow or yellowish hue were classified as yellow (Y). Subsequently, ejaculates were diluted 1:1 (*v*/*v*) with Extendyl diluent (IMV Technologies, L’Aigle, France), placed in a thermobox at 38– 39 °C, and promptly transported to the laboratory.

The samples were analyzed for sperm concentration, motility, viability, and functionality, and centrifuged at 10,000× *g* for 10 min. The obtained plasmas were transferred to separate tubes containing 1:100 of Protease and Phosphatase Inhibitor Cocktail (Sigma-Aldrich, Merck, Burlington, MA, USA). The samples were stored at −80 °C until further analyses.

All biochemical analyses were performed using a UV-Vis DU 800 spectrophotometer (Beckman Coulter, Inc., Brea, CA, USA). All collected seminal plasmas were tested for biochemical parameters in duplicates.

### 4.1. Determination of Total Protein Content

Total protein content was determined using Bradford reagent (Sigma-Aldrich, Merck, Burlington, MA, USA), according to the procedure specified by the manufacturer.

### 4.2. Determination of Alkaline (ALP) and Acid Phosphatase (ACP) Activity

The activity of alkaline phosphatase (ALP) and acid phosphatase (ACP) was determined using the method described by Bessey et al. [87]. To measure ALP activity, 250 µL of an alkaline substrate solution (8 mM disodium phenylphosphate and 56 mM sodium veronal, pH 9.3) and 25 µL of the test sample were added to the tubes. In turn, ACP activity was determined by adding 275 µL of an acidic substrate solution (8 mM disodium phenylphosphate, 40 mM citric acid, and 70 mM sodium citrate; pH 4.9) and 50 µL of the test sample to the tubes. Blank samples were prepared in the same way as the test samples by replacing the sample volume with deionized water. All samples were incubated in a water bath at 37 °C for 30 min. To inhibit the reaction, sodium hydroxide (NaOH) solution was added to the samples: 2.5 mL of 0.02N NaOH was added to ALP samples, and 1 mL of 0.1N NaOH was added to ACP samples. Absorbance was measured against blank samples at 410 nm. Enzyme activity in Bessey–Lowry–Brock (BLB) units was read from the calibration curves and converted to international units using a conversion factor of 16.67.

### 4.3. Determination of Superoxide Dismutase (SOD) Activity

The activity of superoxide dismutase (SOD) in turkey seminal plasmas was determined using a commercial Ransod kit (Randox, Crumlin, UK), according to the manufacturer’s instructions.

### 4.4. Determination of Glutathione Peroxidase (GPx) Activity

Glutathione peroxidase (GPx) activity was determined using a Ransel kit (Randox, Crumlin, UK) according to the manufacturer’s instructions. 

### 4.5. Determination of Catalase (CAT) Activity

Catalase (CAT) activity was measured using a commercial Catalase Assay Kit (Sigma-Aldrich, Merck, Burlington, MA, USA), according to the protocol provided by the manufacturer.

### 4.6. Determination of Glutathione (GSH) Content

The content of reduced glutathione (GSH) was determined using a Bioxytech GSH-400 reagent kit (AOXRE Bio-Sciences, Burlingame, CA, USA), according to the manufacturer’s protocol.

### 4.7. Determination of Malondialdehyde (MDA) Levels

Malondialdehyde (MDA) levels were measured using a Bioxytech MDA-586 kit (AOXRE Bio-Sciences, Burlingame, CA, USA), according to the protocol provided by the manufacturer.

### 4.8. Isolation of Phosphoproteins by Fe^3+^ Ion Affinity Chromatography on a PHOS-Select Iron Affinity Gel Bed

A PHOS-Select Iron Affinity Gel chromatography bed (Sigma-Aldrich, Merck, Burlington, MA, USA) was used to isolate phosphoproteins from seminal fluids. First, 40 µL of the sample was placed in a tube and equilibrated three times with 500 µL of buffer A (250 mM acetic acid, 30% acetonitrile; pH 2.9). After each addition of the buffer, the samples were stirred and centrifuged at 10,000× *g* for 30 s. Then, 500 µL of a sample containing 1 mg of total protein was added to the prepared bed. Seminal plasma was diluted with buffer A. The tubes were placed on a laboratory cradle and stirred at room temperature for 30 min. The tubes were centrifuged at 10,000× *g* for 30 s. The supernatant was removed, and 500 µL of buffer A was added. The samples were centrifuged again. The supernatants were removed, and the bed was washed with 500 µL of deionized water. The tubes were centrifuged again, the supernatant was removed, and 100 µL of buffer B (400 mM ammonia) was added to the bed. The supernatants were stirred, centrifuged, and frozen at −80 °C.

### 4.9. SDS-PAGE Electrophoresis of Seminal Plasma Phosphoproteins

SDS-PAGE separation was performed on 12% polyacrylamide gel [88]. The samples were prepared by combining 10 µL of the solution of the previously isolated phosphoproteins with 2 µL of a lysis buffer (1 M Tris-HCl, 20% SDS, 20% glycerol, 2% β-mercaptoethanol, 2% bromophenol blue; pH 6.8). The obtained solution was loaded into wells. Electrophoresis was performed using a Mini-Protean II Cell apparatus (Bio-Rad Laboratories, Hercules, CA, USA) in a buffer of 50 mM Tris, 250 mM glycine, and 0.5% SDS, pH 8.3. After separation, the gels were stained with Coomassie Brilliant Blue R-250 solution (30% methanol, 5% acetic acid, 0.1% CBB R-250) (Sigma-Aldrich, Merck, Burlington, MA, USA) for 24 hours. The gels were decolorized with 5% methanol and 7% acetic acid. Molecular weight was determined using Precision Plus Protein Standards, and a densitometric analysis of protein gels was performed using Multi Analyst version 1.1. software (Bio-Rad Laboratories, Hercules, CA, USA).

### 4.10. Western Blotting and Immunodetection of Seminal Plasma Phosphoproteins

Phosphoproteins were first subjected to SDS-PAGE separation, and proteins were then electrotransferred to membranes. Biotinylated Molecular Weight Markers (Sigma-Aldrich, Merck, Burlington, MA, USA) were used as the standard proteins during the separation. PVDF membranes (Merck Millipore, Burlington, MA, USA) were used in Western blotting. The membranes were immersed in methanol and placed in a transfer buffer (50 mM Tris, 250 mM glycine, 20% methanol). Proteins were transferred to a semi-dry blotter (Sigma-Aldrich, Merck, Burlington, MA, USA) at a constant current of 1 mA/cm^2^ membrane, applied for one hour.

The membranes were incubated at room temperature for two hours in a washing solution (20 mM Tris, 136 mM NaCl, 0.1% Tween-20; pH 7.5) containing 2% bovine serum albumin (BSA). After incubation, PVDF membranes were washed three times with a wash buffer for 5 min each time. The membranes were then incubated at 4 °C in a wash buffer (50 mL) containing selected biotinylated monoclonal antibodies for at least 12 h on a laboratory cradle. Anti-phosphoserine, anti-phosphothreonine, or anti-phosphotyrosine antibodies were applied in the dilutions recommended by the manufacturer. After incubation, the membranes were washed with wash buffer and incubated for one hour at room temperature in 50 µL of ALP-conjugated streptavidin buffer (Vector Laboratories, Newark, CA, USA). After incubation, the membranes were washed with wash buffer (3 × 5 min), followed by deionized water (1 min). Then, 200 µL of NBT/BCIP Stock Solution mixture (Sigma-Aldrich, Merck, Burlington, MA, USA) was added to 10 mL of an ALP buffer (100 mM Tris, 100 mM NaCl; pH 9.5). The membranes were left in the mixture until the stained fractions achieved the optimal color, after which they were air-dried. The molecular mass of the stained phosphoprotein fractions was determined using Multi Analyst version 1.1. software (Bio-Rad Laboratories, Hercules, CA, USA).

### 4.11. Identification of Seminal Plasma Phosphoproteins by Nano LC-MS/MS

Excised SDS-PAGE gel sections containing phosphoprotein fractions were placed in 50 µL of 100 mM ammonium carbonate and shaken in a thermoblock for 10 min. Then, 50 µL of acetonitrile was added, and the tubes were shaken at 600 rpm for 10 min. The resulting supernatant was removed, and gel fragments were dehydrated by adding 100% acetonitrile and shaking. Acetonitrile was removed, gel fragments were flooded with 50 µL of dithiothreitol solution (DTT) and incubated for 10 min at 90 °C. Next, 50 µL of 5 mM iodoacetamide solution in 100 mM carbonate buffer was added (90 °C, 10 min, 600 rpm). The iodoacetamide solution was removed, and gel sections were dehydrated again using 100% acetonitrile. In the next step, 1.5 pmol of trypsin (Promega, Madison, WI, USA) was added to gel sections and incubated in a carbonate buffer at 37 °C for 12 h. Peptides were extracted with 50 mM carbonate buffer and 5% formic acid solution in 50% acetonitrile. Fractions containing eluted peptides were combined, lyophilized, and dissolved in 30 µL of 4% acetonitrile and 0.1% formic acid solution.

The nano LC-MS/MS analysis was performed using an Ultimate 3000 HPLC/UPLC system connected online to an Exploris 240 mass spectrometer and Thermo Fisher Scientific columns (Waltham, MA, USA). Samples of 5 µL were injected into a system composed of an RP C18 precolumn (0.3 × 5 mm, 5 µm grain diameter) and an RP C18 column (15 cm, 3 µm, 75 µm grain diameter) at a mobile phase flow rate of 300 nL/min. The samples were eluted using a 33-min gradient of solutions A (0.1% fluoroacetic acid in water) and B (0.1% fluoroacetic acid in acetonitrile). The proportion of solution B in the elution gradient was as follows: 8% at the start and after 4 min, 35% after 30 min, 90% after 30 min 30 s and after 31 min 30 s, and 8% after 32 and 33 min.

### 4.12. Functional Analysis of the Identified Phosphoproteins

The gene ontology (GO) enrichment analysis of the genes encoding selected phosphoproteins in turkey seminal plasma was performed in the ShinyGO programme (version 0.77, http://bioinformatics.sdstate.edu/go/, accessed on 1 June 2023). The phosphoproteins present in the mentioned fluid were classified according to their biological functions.

The STRING program (version 11.5, http://string-db.org/, accessed on 1 June 2023) was used to analyze protein–protein interactions between turkey seminal plasma phosphoproteins. The minimum required interaction score was set at ≥0.4 (mean confidence level). All interactions were queried using the *Homo sapiens* database (the largest and most regularly updated gene database).

### 4.13. Statistical Analysis

The statistical analysis was performed using Statistica software (version 13.3., StatSoft, TIBCO Software, Palo Alto, CA, USA). The results were presented as means ± standard errors of the means (SEM). One-way ANOVA and Tukey’s test processed the biochemical parameters of white and yellow seminal plasmas. The parameters related to the optical density of seminal plasma phosphoproteins identified by immunodetection were processed statistically by non-parametric analysis and the Mann–Whitney U test.

## 5. Conclusions

The findings of this study provide new insights into the phosphoproteome composition of turkey seminal plasma. Additionally, they highlight the roles of the identified phosphoproteins and reveal differences between the phosphoproteomes of white and yellow seminal plasma. We suspect that the activity of phosphatases and antioxidant enzymes in semen may have impacted the phosphoproteome profiles of turkey seminal plasmas. The obtained results offer a valuable foundation for future research aimed at analysing the phosphoproteome, particularly in low-quality turkey semen, and identifying potential biomarkers for pathological conditions and/or reduced fertility.

## Figures and Tables

**Table 1:** Biochemical parameters of white and yellow seminal plasmas in turkeys (means ± SEMs).

Parameters	White	Yellow
**Protein content (mg/mL)**	7.63 ± 0.42 ^A^	17.87 ± 0.34 ^B^
**ALP activity (U/mL)**	26.55 ± 2.32 ^B^	75.43 ± 4.89 ^A^
**ACP activity (U/mL)**	6918.98 ± 1284.72 ^b^	11,651.34 ± 1710.60 ^a^
**SOD activity (U/mL)**	2.19 ± 0.14	1.90 ± 0.21
**GPx activity (U/mL)**	0.39 ± 0.02 ^B^	0.65 ± 0.07 ^A^
**CAT activity (µM/min/mL)**	27.64 ± 4.65	29.74 ± 7.92
**GSH content (µM/mL)**	437.82 ± 34.14 ^B^	671.61 ± 47.57 ^A^
**MDA level (µM/mL)**	10.82 ± 1.69 ^B^	18.91 ± 2.08 ^A^

A,B—highly significant differences (*p* ≤ 0.01), a,b—significant differences (*p* ≤ 0.05).

**Table 2:** The mean optical density (OD) values of the fractions selected for identification from white and yellow seminal plasmas in turkeys.

Protein (kDa)	White	Yellow
**106**	0.23	0.28
**76**	0.41	0.26
**69**	0.70	0.83
**41**	0.45	0.49
**31**	0.50	0.69
**30**	0.45	0.48
**29**	0.39	0.41
**28**	0.40	0.46
**27**	0.47	0.45
**26**	0.45	0.48
**20**	0.30	0.34
**16**	0.28	0.24
**11**	0.23	0.19
**7**	0.19	0.24

**Table 3:** The optical density (OD) values of the turkey seminal plasma proteins subjected to phosphorylation on different residues, i.e., serine (SER), threonine (THR), and tyrosine (TYR).

Protein [kDa]	Molecular Weight by Multi-Analyst	Phosphorylated Residue	Type of Semen	Mean	± SEM
**1**	106 kDa	**SER**	**W**	**0.18**	**0.01**
			**Y**	**0.09**	**0.01**
		**THR**	**W**	**0.15**	**0.01**
			**Y**	**0.12**	**0.01**
		**TYR**	**W**	**0.16**	**0.01**
			**Y**	**0.06**	**0.01**
**2**	76 kDa	SER	W	0.24	0.01
			Y	0.27	0.01
		**THR**	**W**	**0.20**	**0.01**
			**Y**	**0.16**	**0.01**
		**TYR**	**W**	**0.27**	**0.01**
			**Y**	**0.11**	**0.01**
**3**	69 kDa	**SER**	**W**	**0.24**	**0.01**
			**Y**	**0.21**	**0.01**
		**THR**	**W**	**0.21**	**0.01**
			**Y**	**0.18**	**0.00**
		**TYR**	**W**	**0.27**	**0.01**
			**Y**	**0.11**	**0.01**
**4**	41 kDa	SER	W	0.23	0.03
			Y	0.26	0.02
		THR	W	0.22	0.03
			Y	0.25	0.03
		**TYR**	**W**	**0.23**	**0.02**
			**Y**	**0.10**	**0.01**
**5**	31 kDa	SER	W	0.24	0.02
			Y	0.22	0.02
		THR	W	0.23	0.01
			Y	0.17	0.01
		**TYR**	**W**	**0.24**	**0.02**
			**Y**	**0.09**	**0.02**
**6**	30 kDa	SER	W	0.24	0.02
			Y	0.24	0.02
		THR	W	0.23	0.01
			Y	0.20	0.01
		**TYR**	**W**	**0.22**	**0.02**
			**Y**	**0.11**	**0.02**
**7**	29 kDa	SER	W	0.48	0.05
			Y	0.34	0.04
		THR	W	0.39	0.04
			Y	0.35	0.06
		**TYR**	**W**	**0.45**	**0.06**
			**Y**	**0.17**	**0.04**
**8**	28 kDa	SER	W	0.24	0.06
			Y	0.20	0.04
		THR	W	0.20	0.03
			Y	0.21	0.05
		**TYR**	**W**	**0.24**	**0.04**
			**Y**	**0.11**	**0.03**
**9**	27 kDa	SER	W	0.16	0.02
			Y	0.17	0.01
		THR	W	0.14	0.01
			Y	0.16	0.01
		**TYR**	**W**	**0.15**	**0.01**
			**Y**	**0.09**	**0.02**
**10**	26 kDa	SER	W	0.17	0.02
			Y	0.18	0.01
		THR	W	0.14	0.01
			Y	0.16	0.01
		**TYR**	**W**	**0.15**	**0.01**
			**Y**	**0.10**	**0.02**
**11**	20 kDa	SER	W	0.08	0.01
			Y	0.12	0.02
		THR	W	0.11	0.01
			Y	0.13	0.01
		TYR	W	0.06	0.02
			Y	0.10	0.01
**12**	16 kDa	SER	W	0.09	0.01
			Y	0.10	0.01
		THR	W	0.10	0.01
			Y	0.12	0.02
		TYR	W	0.06	0.00
			Y	0.08	0.01
**13**	11 kDa	SER	W	0.06	0.01
			Y	0.06	0.01
		THR	W	0.07	0.00
			Y	0.07	0.01
		**TYR**	**W**	**0.05**	**0.01**
			**Y**	**0.03**	**0.00**
**14**	7 kDa	SER	W	0.06	0.00
			Y	0.05	0.01
		THR	W	0.09	0.01
			Y	0.07	0.01
		**TYR**	**W**	**0.05**	**0.01**
			**Y**	**0.02**	**0.00**

OD values given **in bold** are statistically significant (*p* ≤ 0.05).

## Data Availability

The data that support the findings of this study are available on request from the corresponding author, A.O.

## Supplementary Materials

The following supporting information can be downloaded at: https://www.mdpi.com/article/10.3390/ijms25189941/s1.
