# Supplementary material for: A Comparison of White and Yellow Seminal Plasma Phosphoproteomes Obtained from Turkey (Meleagris gallopavo) Semen
Source: Int J Mol Sci. 2024 Sep 14;25(18):9941. doi: 10.3390/ijms25189941 (PMC11432639; doi:10.3390/ijms25189941)
Supplement: Supplementary file 1 [file ijms-25-09941-s001.zip › ijms-3171666-supplementary.pdf]

Table S1. Phosphoproteins identified in turkey seminal plasma by SDS-PAGE and nano LC-MS/MS.

No	Identified protein	Gene	Species	Molecular weight in Multi-Analyst	Molecular weight (Da)	Number of compatible peptides	Sequence coverage [%]	Score
**1**	Albumin	ALB	*Gallus gallus*	106 kDa	71868	126	47	2578
	Cysteine protease ATG4B	ATG4B	*Gallus gallus*		45186	20	19	969
	Fibronectin	FN1	*Gallus gallus*		276669	12	5	514
	Myosin-9	MYH9	*Gallus gallus*		227446	5	4	271
	Collagen alpha-1(XII) chain	COL12A1	*Gallus gallus*		341740	6	2	196
	Ovotransferrin	TRFE	*Gallus gallus*		79551	14	21	181
	Cation-transporting ATPase	ATP13A4	*Gallus gallus*		135682	6	4	155
	Fatty acid synthase	FASN	*Gallus gallus*		277630	3	1	91
	Low-density lipoprotein receptor-related protein 1	LRP1	*Gallus gallus*		525788	1	1	83
	Receptor-type tyrosine-protein phosphatase eta	PTPRJ	*Gallus gallus*		155087	2	1	72
	Proto-oncogene tyrosine-protein kinase ROS	ROS1	*Gallus gallus*		263419	2	1	60
**2**	Albumin	ALB	*Gallus gallus*	76 kDa	71868	583	58	5722
	Elongation factor 2	EEF2	*Gallus gallus*		96343	18	23	720
	Cysteine protease ATG4B	ATG4B	*Gallus gallus*		45186	12	19	589
	Fibronectin	FN1	*Gallus gallus*		276669	8	4	237
	Collagen alpha-1(XII) chain	COL12A1	*Gallus gallus*		341740	3	1	153
	Protein NEL	NEL	*Gallus gallus*		96096	7	6	151
	Alpha-actinin-1	ACTN1	*Gallus gallus*		103610	4	2	88
	Alpha-actinin-4	ACTN4	*Gallus gallus*		104712	4	2	88
	Ovotransferrin	TRFE	*Gallus gallus*		79551	12	22	66
	Cytoplasmic aconitate hydratase	ACO1	*Gallus gallus*		98639	1	2	61
**3**	Ovotransferrin	TRFE	*Gallus gallus*	69 kDa	79551	437	71	2016
	Albumin	ALB	*Gallus gallus*		71868	76	35	1534
	Transferrin receptor protein 1	TFRC	*Gallus gallus*		85890	3	20	146
	Cysteine protease ATG4B	ATG4B	*Gallus gallus*		45186	2	15	112
	Endoplasmic reticulum chaperone BiP	HSPA5	*Gallus gallus*		72088	1	2	68
	Sulfhydryl oxidase 1	QSOX1	*Gallus gallus*		83939	1	7	63
**4**	Albumin	ALB	*Gallus gallus*	41 kDa	71868	83	26	3346
	Ovotransferrin	TRFE	*Gallus gallus*		79551	28	17	1187

Glyceraldehyde-3-phosphate dehydrogenase	GAPDH	*Gallus gallus*	41 kDa	35909	25	45	1005
Glyceraldehyde-3-phosphate dehydrogenase	GAPDH	*Meleagris gallopavo*		25065	25	45	1005
Creatine kinase B-type	CKB	*Gallus gallus*		43129	15	37	809
Sulfhydryl oxidase 1	QSOX1	*Gallus gallus*		83939	22	11	792
Malate dehydrogenase, cytoplasmic	MDH1	*Gallus gallus*		36748	23	38	789
Astacin-like metalloendopeptidase	ASTL	*Gallus gallus*		46929	18	15	592
Annexin A2	ANXA2	*Gallus gallus*		38901	8	16	333
Gelsolin	GSN	*Gallus gallus*		86120	8	5	332
Alpha-1-acid glycoprotein 2	ORM2	*Meleagris gallopavo*		22369	2	33	203
Acrosin	ACR	*Meleagris gallopavo*		38724	3	9	188
L-lactate dehydrogenase A chain	LDHA	*Gallus gallus*		36776	4	9	182
L-lactate dehydrogenase B chain	LDHB	*Gallus gallus*		36694	4	8	174
SPARC	SPARC	*Gallus gallus*		34894	3	9	166
Elongation factor 1-alpha 1	EEF1A	*Gallus gallus*		50467	5	7	165
Charged multivesicular body protein 4b	CHMP4B	*Gallus gallus*		25140	3	9	137
Cysteine protease ATG4B	ATG4B	*Gallus gallus*		45186	3	6	130
Tubulin beta chain	TBB1<br>TBB2<br>TBB3<br>TBB4<br>TBB5<br>TBB6<br>TBB7	*Gallus gallus*		50333<br>50377<br>50285<br>50844<br>50395<br>50692<br>50095	5	4	122
Actin	ACTA1<br>ACTA2<br>ACTB<br>ACTC<br>ACTG<br>ACTG2<br>ACT5	*Gallus gallus*		42366<br>42367<br>42052<br>42334<br>42108<br>42249<br>42151	2	4	120
Heat shock cognate 71 kDa protein	HSPA8	*Gallus gallus*		71011	3	6	113
Heat shock protein HSP 90	HSP90AA1<br>HSP90AB1	*Gallus gallus*		84406<br>83717	3	3<br>3	99
Aspartate aminotransferase, cytoplasmic	GOT1	*Gallus gallus*		46134	2	6	93
Creatine kinase M-type	CKM	*Gallus gallus*		43529	1	4	87

	Neuronal growth regulator 1	NEGR1	*Gallus gallus*	41 kDa	38434	1	3	64
	Tubulin alpha-2 chain	TBA2	*Gallus gallus*		50450	1	3	63
	F-actin-capping protein subunit alpha-1	CAPZA1	*Gallus gallus*		33110	1	3	59
	F-actin-capping protein subunit alpha-2	CAPZA2	*Gallus gallus*		32939	1	3	59
	Clusterin	CLU	*Coturnix japonica*		52395	1	5	57
	Pyruvate kinase PKM	PKM	*Gallus gallus*		58434	1	2	56
	Protein NEL	NEL	*Gallus gallus*		96096	1	1	56
	Aminopeptidase Ey	ANPEP	*Gallus gallus*		109406	1	1	54
	Radixin	RDX	*Gallus gallus*		68626	1	1	52
	Apolipoprotein AI	APOA1	*Gallus gallus*		30661	1	4	50
	Collagen alpha-1(XII) chain	COL12A1	*Gallus gallus*		341740	1	1	50
**5**	Albumin	ALB	*Gallus gallus*	31 kDa	71868	27	32	996
	Sulfhydryl oxidase 1	QSOX1	*Gallus gallus*		83939	17	9	711
	Creatine kinase B-type	CKB	*Gallus gallus*		43129	11	19	595
	Ovotransferrin	TRFE	*Gallus gallus*		79551	9	8	343
	Astacin-like metalloendopeptidase	ASTL	*Gallus gallus*		46929	9	6	278
	SPARC	SPARC	*Gallus gallus*		34894	4	9	224
	Fibronectin	FN1	*Gallus gallus*		276669	3	2	220
	Neuronal growth regulator 1	NEGR1	*Gallus gallus*		38434	7	17	215
	Annexin A2	ANXA2	*Gallus gallus*		38901	4	13	210
	Golgi apparatus protein 1	GLG1	*Gallus gallus*		43386	2	4	189
	Heat shock cognate 71 kDa protein	HSPA8	*Gallus gallus*		71011	3	4	112
	Heat shock protein HSP 90	HSP90AA1<br>HSP90AB1	*Gallus gallus*		84406<br>83717	3	3	105
	Gelsolin	GSN	*Gallus gallus*		86120	2	1	101
	Aminopeptidase Ey	ANPEP	*Gallus gallus*		109406	3	2	99
	Malate dehydrogenase, cytoplasmic	MDH1	*Gallus gallus*		36748	1	5	82
	Protein NEL	NEL	*Gallus gallus*		96096	3	1	78
	Collagen alpha-1(XII) chain	COL12A1	*Gallus gallus*		341740	2	1	78
	Creatine kinase M-type	CKM	*Gallus gallus*		43529	1	4	77
	Elongation factor 1-alpha 1	EEF1A	*Gallus gallus*		50467	2	2	70
	Glyceraldehyde-3-phosphate dehydrogenase	GAPDH	Gallus gallus		35909	1	4	58
	Glyceraldehyde-3-phosphate dehydrogenase	GAPDH	*Meleagris gallopavo*		25065	1	4	58
	Tubulin beta-1 chain	TBB1	*Gallus gallus*		50333	1	3	52
	Tubulin beta-2 chain	TBB2	*Gallus gallus*		50377	1	3	52

	Tubulin beta-3 chain	TBB3	*Gallus gallus*	31 kDa	50285	1	3	52
	Tubulin beta-7 chain	TBB7	*Gallus gallus*		50095	1	3	52
	Pyruvate kinase PKM	PKM	*Gallus gallus*		58434	1	2	48
	Actin	ACTA1<br>ACTA2<br>ACTB<br>ACTC<br>ACTG<br>ACTG2<br>ACT5	*Gallus gallus*		42366<br>42367<br>42052<br>42334<br>42108<br>42249<br>42151	1	5	71
	Cysteine protease ATG4B	ATG4B	*Gallus gallus*		45186	1	6	40
**6**	Albumin	ALB	*Gallus gallus*	30 kDa	71868	66	22	2198
	Creatine kinase M-type	CKM	*Gallus gallus*		43529	7	13	390
	Astacin-like metalloendopeptidase	ASTL	*Gallus gallus*		46929	9	10	303
	Ovotransferrin	TRFE	*Gallus gallus*		79551	6	8	282
	Creatine kinase B-type	CKB	*Gallus gallus*		43129	4	9	196
	SPARC	SPARC	*Gallus gallus*		34894	3	9	170
	T-complex protein 1 subunit theta	CCT8	*Gallus gallus*		60017	2	2	147
	Voltage-dependent anion-selective channel protein 2	VDAC2	*Meleagris gallopavo*		30162	2	3	130
	Sulfhydryl oxidase 1	QSOX1	*Gallus gallus*		83939	2	1	102
	1-phosphatidylinositol 4,5-bisphosphate phosphodiesterase zeta-1	PLCZ1	*Gallus gallus*		73285	2	4	100
	Glyceraldehyde-3-phosphate dehydrogenase	GAPDH	*Gallus gallus*		35909	2	4	86
	Glyceraldehyde-3-phosphate dehydrogenase	GAPDH	*Meleagris gallopavo*		25065	2	4	86
	Malate dehydrogenase, cytoplasmic	MDH1	*Gallus gallus*		36748	1	3	86
	Acrosin	ACR	*Meleagris gallopavo*		38724	1	4	82
	Annexin A1	ANXA1	*Gallus gallus*		14446	1	4	72
	Transthyretin	TTR	*Gallus gallus*		16356	1	10	66
	Frizzled-7	FZD7	*Gallus gallus*		64081	1	5	45
**7**	Albumin	ALB	*Gallus gallus*	29 kDa	71868	31	28	794
	Astacin-like metalloendopeptidase	ASTL	*Gallus gallus*		46929	18	18	362
	Cysteine protease ATG4B	ATG4B	*Gallus gallus*		45186	6	11	361
	1-phosphatidylinositol 4,5-bisphosphate phosphodiesterase zeta-1	PLCZ1	*Gallus gallus*		73285	9	8	240
	Ig lambda chain C region	LAC	*Gallus gallus*		11525	2	11	96

	Voltage-dependent anion-selective channel protein 2	VDAC2	*Meleagris gallopavo*		30162	2	3	86
	Ovotransferrin	TRFE	*Gallus gallus*		79551	1	22	67
**8**	Albumin	ALB	*Gallus gallus*	28 kDa	71868	61	54	2571
	Creatine kinase B-type	CKB	*Gallus gallus*		43129	16	28	951
	Sulfhydryl oxidase 1	QSOX1	*Gallus gallus*		83939	8	9	322
	Gelsolin	GSN	*Gallus gallus*		86120	6	5	298
	Acrosin	ACR	*Meleagris gallopavo*		38724	6	11	282
	Ovotransferrin	TRFE	*Gallus gallus*		79551	6	7	277
	Astacin-like metalloendopeptidase	ASTL	*Gallus gallus*		46929	7	14	269
	Glyceraldehyde-3-phosphate dehydrogenase	GAPDH	*Gallus gallus*		35909	6	9	267
	Glyceraldehyde-3-phosphate dehydrogenase	GAPDH	*Meleagris gallopavo*		25065	6	9	267
	14-3-3 protein zeta	YWHAZ	*Gallus gallus*		27929	5	25	229
	Phosphoglycerate mutase 1	PGAM1	*Gallus gallus*		29051	5	22	203
	Ig lambda chain C region	LAC	*Gallus gallus*		11525	4	11	173
	14-3-3 protein theta	YWHAQ	*Gallus gallus*		28050	3	21	168
	Tubulin alpha chain	TBA1<br>TBA8	*Gallus gallus*		46385<br>36651	4	4	149
	Actin	ACTB<br>ACTG<br>ACT5	*Gallus gallus*		42052<br>42108<br>42151	3	9	143
	Malate dehydrogenase, cytoplasmic	MDH1	*Gallus gallus*		36748	2	3	123
	Fibronectin	FN1	*Gallus gallus*		276669	3	1	114
	Heat shock cognate 71 kDa protein	HSPA8	*Gallus gallus*		71011	2	5	110
	14-3-3 protein epsilon	YWHAE	*Gallus gallus*		29326	2	11	102
	Tubulin beta chain	TBB1<br>TBB2<br>TBB3<br>TBB4<br>TBB5<br>TBB6<br>TBB7	*Gallus gallus*	28 kDa	50333<br>50377<br>50285<br>50844<br>50395<br>50692<br>50095	3	10	97
	Collagen alpha-1(XII) chain	COL12A1	*Gallus gallus*		341740	2	1	85
	Creatine kinase M-type	CKM	*Gallus gallus*		43529	1	4	85
	1-phosphatidylinositol 4,5-bisphosphate phosphodiesterase zeta-1	PLCZ1	*Gallus gallus*		73285	1	2	73

	Protein NEL	NEL	*Gallus gallus*		96096	2	1	70
	Elongation factor 1-alpha 1	EEF1A	*Gallus gallus*		50467	2	2	70
	Platelet-activating factor acetylhydrolase IB subunit alpha2	PAFAH1B2	*Gallus gallus*		25665	1	3	56
	Peroxiredoxin-6	PRDX6	*Gallus gallus*		25075	1	3	56
	SPARC	SPARC	*Gallus gallus*		34894	1	4	50
**9**	Albumin	ALB	*Gallus gallus*	27 kDa	71868	43	59	1660
	Ovotransferrin	TRFE	*Gallus gallus*		79551	10	6	352
	Gelsolin	GSN	*Gallus gallus*		86120	7	5	342
	Ig lambda chain C region	LAC	*Gallus gallus*		11525	7	11	270
	Acrosin	ACR	*Meleagris gallopavo*		38724	7	9	265
	Astacin-like metalloendopeptidase	ASTL	*Gallus gallus*		46929	7	6	234
	Sulfhydryl oxidase 1	QSOX1	*Gallus gallus*		83939	5	5	222
	Apolipoprotein AI	APOA1	*Gallus gallus*		30661	8	12	2110
	Creatine kinase B-type	CKB	*Gallus gallus*		43129	5	9	195
	Ferritin heavy chain	FTH	*Gallus gallus*		21249	8	18	190
	Tubulin beta chain	TBB1<br>TBB2<br>TBB3<br>TBB4<br>TBB5<br>TBB6<br>TBB7	*Gallus gallus*		50333<br>50377<br>50285<br>50844<br>50395<br>50692<br>50095	5	11	148
	Collagen alpha-1(XII) chain	COL12A1	*Gallus gallus*		341740	1	1	100
	Glyceraldehyde-3-phosphate dehydrogenase	GAPDH	*Gallus gallus*		35909	2	4	70
	Glyceraldehyde-3-phosphate dehydrogenase	GAPDH	*Meleagris gallopavo*		25065	2	4	70
	Heat shock protein HSP 90	HSP90AA1<br>HSP90AB1	*Gallus gallus*		84406<br>83717	3	1	105
	Peroxiredoxin-1	PRDX1	*Gallus gallus*		22529	2	5	65
	Peroxiredoxin-6	PRDX6	*Gallus gallus*	27 kDa	25075	2	8	65
	Peptidyl-prolyl cis-trans isomerase B	PPIB	*Gallus gallus*		22456	7	23	232
	Transthyretin	TTR	*Gallus gallus*		16356	1	9	56
	Protein NEL	NEL	*Gallus gallus*		96096	1	6	55
	SPARC	SPARC	*Gallus gallus*		34894	1	9	54

	1-phosphatidylinositol 4,5-bisphosphate phosphodiesterase zeta-1	PLCZ1	*Gallus gallus*		73285	1	12	54
	Actin	ACTA1<br>ACTA2<br>ACTB<br>ACTC<br>ACTG<br>ACTG2<br>ACT5	*Gallus gallus*		42366<br>42367<br>42052<br>42334<br>42108<br>42249<br>42151	1	8	71
	GTPase HRas	HRAS	*Gallus gallus*		21694	1	4	47
	GTPase NRas	NRAS			21550	1	4	47
	Neuronal growth regulator 1	NEGR1	*Gallus gallus*		38434	1	6	46
**10**	Albumin	ALB	*Gallus gallus*		71868	71	63	2910
	Creatine kinase B-type	CKB	*Gallus gallus*		43129	8	15	271
	Apolipoprotein A-I	APOA1	*Gallus gallus*		30661	11	24	264
	Ig lambda chain C region	LAC	*Gallus gallus*		11525	5	8	213
	Astacin-like metalloendopeptidase	ASTL	*Gallus gallus*		46929	11	5	264
	Immunoglobulin lambda constant 1	IGLC1	*Meleagris gallopavo*	26 kDa	27892	2	18	187
	Tubulin beta-3 chain	TBB3	*Gallus gallus*		50285	6	8	176
	Tubulin beta-5 chain	TBB5	*Gallus gallus*		50395	6	8	176
	Phosphoglycerate mutase 1	PGAM1	*Gallus gallus*		29051	2	15	167
	Ovotransferrin	TRFE	*Gallus gallus*		79551	4	3	163
	Acrosin	ACR	*Meleagris gallopavo*		38724	2	9	137
	Glyceraldehyde-3-phosphate dehydrogenase	GAPDH	*Gallus gallus*		35909	3	4	129
	Glyceraldehyde-3-phosphate dehydrogenase	GAPDH	*Meleagris gallopavo*		25065	3	4	129
	Immunoglobulin lambda constant 3	IGLC3	*Meleagris gallopavo*		11567	2	8	118
	Creatine kinase M-type	CKM	*Gallus gallus*		43529	2	7	98
	Actin	ACTA1<br>ACTA2<br>ACTB<br>ACTC<br>ACTG<br>ACTG2<br>ACT5	*Gallus gallus*	26 kDa	42366<br>42367<br>42052<br>42334<br>42108<br>42249<br>42151	1	4	71
	Aspartate aminotransferase, cytoplasmic	GOT1	*Gallus gallus*		46134	1	3	69

	SPARC	SPARC	*Gallus gallus*		34894	1	4	57
**11**	Albumin	ALB	*Gallus gallus*	20 kDa	71868	52	58	1967
	Ovotransferrin	TRFE	*Gallus gallus*		79551	21	13	891
	Creatine kinase B-type	CKB	*Gallus gallus*		43129	18	23	881
	Acrosin	ACR	*Meleagris gallopavo*		38724	8	15	594
	Astacin-like metalloendopeptidase	ASTL	*Gallus gallus*		46929	15	13	492
	Gelsolin	GSN	*Gallus gallus*		86120	10	7	431
	Sulfhydryl oxidase 1	QSOX1	*Gallus gallus*		83939	12	7	405
	Ferritin heavy chain	FTH	*Gallus gallus*		21249	16	25	397
	Tubulin beta-4 chain	TBB4	*Gallus gallus*		50844	15	17	382
	Apolipoprotein A-I	APOA1	*Gallus gallus*		30661	14	17	358
	Tubulin beta-5 chain	TBB5	*Gallus gallus*		50395	12	15	308
	Ig lambda chain C region	LAC	*Gallus gallus*		11525	7	11	288
	1-phosphatidylinositol 4,5-bisphosphate phosphodiesterase zeta-1	PLCZ1	*Gallus gallus*		73285	7	8	252
	Peptidyl-prolyl cis-trans isomerase B	PPIB	*Gallus gallus*		22456	7	23	232
	Transthyretin	TTR	*Gallus gallus*		16356	7	50	204
	Soluble scavenger receptor cysteine-rich domain-containing protein SSC5D	SSC5D	*Meleagris gallopavo*		54032	1	25	201
	Heat shock protein HSP 90	HSP90AA1	*Gallus gallus*		84406	6	5	192
	Heat shock cognate 71 kDa protein	HSPA8	*Gallus gallus*		71011	6	9	187
	Proteasome subunit beta type-5	PSMB5	*Gallus gallus*		27256	4	9	159
	Collagen alpha-1(XII) chain	COL12A1	*Gallus gallus*		341740	3	1	156
	Heat shock 70 kDa protein	HSP70	*Gallus gallus*		69936	3	6	156
	Actin	ACTA1<br>ACTA2<br>ACTB<br>ACTC<br>ACTG<br>ACTG2<br>ACT5	*Gallus gallus*		42366<br>42367<br>42052<br>42334<br>42108<br>42249<br>42151	2	4	116
	Glyceraldehyde-3-phosphate dehydrogenase	GAPDH	*Gallus gallus*		35909	2	4	104
	Glyceraldehyde-3-phosphate dehydrogenase	GAPDH	*Meleagris gallopavo*		25065	2	4	104
	Peroxiredoxin-6	PRDX6	*Gallus gallus*		25075	4	8	100
	Carbonic anhydrase 2	CA2	*Gallus gallus*		29388	2	4	89

	Malate dehydrogenase, cytoplasmic	MDH1	*Gallus gallus*	20 kDa	36748	1	3	86
	Triosephosphate isomerase	TPI1	*Gallus gallus*		26832	2	6	80
	Ras-related protein Rap-1b	RAP1B	*Gallus gallus*		21040	1	6	80
	Peroxiredoxin-1	PRDX1	*Gallus gallus*		22529	2	5	75
	Phosphoglycerate kinase	PGK	*Gallus gallus*		45087	1	3	65
	Zona pellucida-binding protein 1	ZPBP1	*Gallus gallus*		36910	2	3	64
	SPARC	SPARC	*Gallus gallus*		34894	1	5	64
	Elongation factor 1-alpha 1	EEF1A	*Gallus gallus*		50467	1	2	55
	Creatine kinase M-type	CKM	*Gallus gallus*		43529	1	4	54
	14-3-3 protein:<br>Beta<br>Epsilon<br>Gamma<br>Theta<br>Zeta	 YWHAB<br>YWHAE<br>YWHAG<br>YWHAQ<br>YWHAZ	*Gallus gallus*		 28004<br>29326<br>28384<br>28050<br>27929	1	6	51
	GTPase HRas<br>GTPase NRas	HRAS<br>NRAS	*Gallus gallus*		21694<br>21550	1	4	47
	Fructose-bisphosphate aldolase C	ALDOC	*Gallus gallus*		14543	1	6	49
	Cell division control protein 42 homolog	CDC42	*Gallus gallus*		21601	1	10	45
**12**	Albumin	ALB	*Gallus gallus*	16 kDa	71868	71	57	2803
	Creatine kinase B-type	CKB	*Gallus gallus*		43129	16	25	894
	Ovotransferrin	TRFE	*Gallus gallus*		79551	18	14	714
	Acrosin	ACR	*Meleagris gallopavo*		38724	6	15	450
	Sulfhydryl oxidase 1	QSOX1	*Gallus gallus*		83939	11	7	378
	Astacin-like metalloendopeptidase	ASTL	*Gallus gallus*		46929	11	11	336
	Gelsolin	GSN	*Gallus gallus*		86120	8	5	325
	Glyceraldehyde-3-phosphate dehydrogenase	GAPDH	*Gallus gallus*		35909	9	12	311
	Glyceraldehyde-3-phosphate dehydrogenase	GAPDH	*Meleagris gallopavo*		25065	9	12	311
	Ras-related protein Rap-1b	RAP1B	*Gallus gallus*		21040	9	13	303
	Collagen alpha-1(XII) chain	COL12A1	*Gallus gallus*		341740	11	2	302
	Cell division control protein 42 homolog	CDC42	*Gallus gallus*		21601	8	25	225
	Ig lambda chain C region	LAC	*Gallus gallus*		11525	5	11	194
	SPARC	SPARC	*Gallus gallus*		34894	3	5	192
	Apolipoprotein A-I	APOA1	*Gallus gallus*		30661	7	12	191
	Heat shock 70 kDa protein	HSP70	*Gallus gallus*		69936	7	10	181

Heat shock cognate 71 kDa protein	HSPA8	*Gallus gallus*		71011	5	8	169
Aspartate aminotransferase, cytoplasmic	GOT1	*Gallus gallus*		46134	3	6	135
Peroxiredoxin-6	PRDX6	*Gallus gallus*		25075	4	15	124
Annexin A2	ANXA2	*Gallus gallus*		38901	3	4	116
Peroxiredoxin-1	PRDX1	*Gallus gallus*	16 kDa	22529	4	18	112
Malate dehydrogenase, cytoplasmic	MDH1	*Gallus gallus*		36748	2	9	107
Actin	ACTA1<br>ACTA2<br>ACTB<br>ACTC<br>ACTG<br>ACTG2<br>ACT5	*Gallus gallus*		42366<br>42367<br>42052<br>42334<br>42108<br>42249<br>42151	2	4	106
Tubulin beta-5 chain	TBB5	*Gallus gallus*		50395	2	6	103
1-phosphatidylinositol 4,5-bisphosphate phosphodiesterase zeta-1	PLCZ1	*Gallus gallus*		73285	3	2	96
14-3-3 protein zeta	YWHAZ	*Gallus gallus*		27929	1	7	94
Ubiquitin-ribosomal protein eS31 fusion protein	RPS27A	*Gallus gallus*		18310	1	11	93
Polyubiquitin-B	UBB	*Gallus gallus*		34348	1	5	93
Na^+/H^+ exchange regulatory cofactor NHE-RF	SLC9A3R1	*Gallus gallus*		36011	2	4	92
Protein/nucleic acid deglycase DJ-1	PARK7	*Gallus gallus*		20159	1	8	82
Ras-related protein Rab-10	RAB10	*Gallus gallus*		22763	2	5	80
Triosephosphate isomerase	TPI1	*Gallus gallus*		26832	2	10	80
Carbonic anhydrase 2	CA2	*Gallus gallus*		29388	2	4	75
Heat shock protein HSP 90	HSP90AA1<br>HSP90AB1	*Gallus gallus*		84406<br>83717	2	1	73
GTPase HRas<br>GTPase NRas	HRAS<br>NRAS	*Gallus gallus*		21694<br>21550	1<br>1	6<br>6	47<br>47
Protein NEL	NEL	*Gallus gallus*		96096	2	1	68
Ras-related protein Rab-2A	RAB2A	*Gallus gallus*		23678	1	8	68
Neuronal growth regulator 1	NEGR1	*Gallus gallus*		38434	2	3	67
Transthyretin	TTR	*Gallus gallus*		16356	1	8	61
Receptor-type tyrosine-protein phosphatase gamma	PTPRG	*Gallus gallus*	16 kDa	160750	1	3	57
Pyruvate kinase PKM	PKM	*Gallus gallus*		58434	1	2	55
Annexin A1	ANXA1	*Gallus gallus*		14446	1	4	52

	Elongation factor 1-alpha 1	EEF1A	*Gallus gallus*		50467	1	2	51
	UMP/CMP kinase	CMPK	*Gallus gallus*		22386	1	6	51
	T-complex protein 1 subunit zeta	CCT6	*Gallus gallus*		58008	1	2	50
	GTP-binding nuclear protein Ran	RAN	*Gallus gallus*		24583	1	6	49
	Ras-related protein Rab-5B	RAB5B	*Gallus gallus*		23828	1	6	45
**13**	Albumin	ALB	*Gallus gallus*		71868	56	57	2146
	Creatine kinase B-type	CKB	*Gallus gallus*		43129	10	16	514
	Acrosin	ACR	*Meleagris gallopavo*		38724	5	9	361
	Tubulin beta-3 chain	TBB3	*Gallus gallus*		50285	9	17	342
	Transthyretin	TTR	*Gallus gallus*		16356	8	35	333
	Astacin-like metalloendopeptidase	ASTL	*Gallus gallus*		46929	9	6	307
	Apolipoprotein A-I	APOA1	*Gallus gallus*		30661	13	16	285
	Creatine kinase M-type	CKM	*Gallus gallus*		43529	4	5	276
	Ig lambda chain C region	LAC	*Gallus gallus*		11525	5	11	234
	Tubulin beta-5 chain	TBB5	*Gallus gallus*		50395	7	12	216
	Tubulin beta-4 chain	TBB4	*Gallus gallus*		50844	6	10	192
	Retinol-binding protein 4	RBP4	*Gallus gallus*	11 kDa	22843	5	25	188
	Ovotransferrin	TRFE	*Gallus gallus*		79551	3	3	134
	SPARC	SPARC	*Gallus gallus*		34894	2	9	121
	Aspartate aminotransferase, cytoplasmic	GOT1	*Gallus gallus*		46134	3	6	98
	Avidin	AVD	*Gallus gallus*		16872	2	7	91
	Calmodulin	CALM<br>CCM1	*Gallus gallus*		16827<br>16990	1	10	82
	ADP-ribosylation factor 5	ARF5	*Gallus gallus*		20581	1	6	74
	Malate dehydrogenase, cytoplasmic	MDH1	*Gallus gallus*		36748	2	3	107
	Ferritin heavy chain	FTH	*Gallus gallus*		21249	1	6	64
	Cathepsin D	CTSD	*Gallus gallus*		43726	1	2	51
	Metalloproteinase inhibitor 2	TIMP2	*Gallus gallus*	11 kDa	24981	1	6	48
**14**	Albumin	ALB	*Gallus gallus*		71868	71	59	2878
	Transthyretin	TTR	*Gallus gallus*		16356	56	67	2142
	Apolipoprotein AI	APOA1	*Gallus gallus*		30661	23	23	535
	Ig lambda chain C region	LAC	*Gallus gallus*	7 kDa	11525	4	11	189
	Tubulin beta-3 chain	TBB3	*Gallus gallus*		50285	8	11	189
	Acrosin	ACR	*Meleagris gallopavo*		38724	2	9	113
	Creatine kinase B-type	CKB	*Gallus gallus*		43129	4	2	109

Astacin-like metalloendopeptidase	ASTL	*Gallus gallus*	46929	2	2	91
Heat shock protein HSP 90	HSP90AA1<br>HSP90AB1	*Gallus gallus*	84406<br>83717	2	9	86
Heat shock cognate 71 kDa protein	HSPA8	*Gallus gallus*	71011	1	5	84
Glyceraldehyde-3-phosphate dehydrogenase	GAPDH	*Gallus gallus*	35909	2	4	78
Glyceraldehyde-3-phosphate dehydrogenase	GAPDH	*Meleagris gallopavo*	25065	2	4	78
Aspartate aminotransferase, cytoplasmic	GOT1	*Gallus gallus*	46134	2	4	65
Ovotransferrin	TRFE	*Gallus gallus*	79551	1	1	61
Ferritin heavy chain	FTH	*Gallus gallus*	21249	1	6	53
Retinol-binding protein 4	RBP4	*Gallus gallus*	22843	1	5	48
Gelsolin	GSN	*Gallus gallus*	86120	1	1	47
Nucleoside diphosphate kinase	NDK	*Gallus gallus*	17448	1	11	46
